# SAR Analysis of Small Molecules Interfering with Energy-Metabolism in *Mycobacterium tuberculosis*

**DOI:** 10.3390/ph13090227

**Published:** 2020-08-31

**Authors:** Federico Appetecchia, Sara Consalvi, Cristina Scarpecci, Mariangela Biava, Giovanna Poce

**Affiliations:** Department of Chemistry and Technologies of Drug, Sapienza University of Rome, piazzale A. Moro 5, 00185 Rome, Italy; federico.appetecchia@uniroma1.it (F.A.); sara.consalvi@uniroma1.it (S.C.); cristina.scarpecci@uniroma1.it (C.S.); mariangela.biava@uniroma1.it (M.B.)

**Keywords:** tuberculosis, drug discovery, antimicrobials, antimycobacterials, energy-metabolism

## Abstract

Tuberculosis remains the world’s top infectious killer: it caused a total of 1.5 million deaths and 10 million people fell ill with TB in 2018. Thanks to TB diagnosis and treatment, mortality has been falling in recent years, with an estimated 58 million saved lives between 2000 and 2018. However, the emergence of multidrug-resistant (MDR) and extensively drug-resistant (XDR) *Mtb* strains is a major concern that might reverse this progress. Therefore, the development of new drugs acting upon novel mechanisms of action is a high priority in the global health agenda. With the approval of bedaquiline, which targets mycobacterial energy production, and delamanid, which targets cell wall synthesis and energy production, the energy-metabolism in *Mtb* has received much attention in the last decade as a potential target to investigate and develop new antimycobacterial drugs. In this review, we describe potent anti-mycobacterial agents targeting the energy-metabolism at different steps with a special focus on structure-activity relationship (SAR) studies of the most advanced compound classes.

## 1. Introduction

Tuberculosis (TB) is the leading cause of death from a single infectious agent worldwide, claiming 1.5 million deaths (including 251 000 people with HIV) and 10 million new cases in 2018 [1]. This success mainly depends on the ability of *Mycobacterium tuberculosis* (*Mtb*) to survive within the host, switching between active and latent disease states, and evading the immune system defenses. Thanks to TB diagnosis and treatment, mortality has been falling in recent years, with an estimated 58 million saved lives between 2000 and 2018. However, the emergence of multidrug-resistant (MDR) and extensively drug-resistant (XDR) *Mtb* strains is a major concern that might reverse this progress. The World Health Organization (WHO) estimates that in 2018 there were 484,000 new cases with resistance to rifampicin (RIF) (RR-TB), the most effective first-line drug, of which 78% had MDR-TB, defined as TB that is resistant to isoniazid (INH) and RIF. A total of 8.5% of MDR-TB cases had XDR-TB, classified as being resistant to INH and RIF, in addition to any fluoroquinolone and injectable second-line drugs [1]. Therefore, the development of new drugs acting upon novel mechanisms of action is a high priority in the global health agenda.

TB drug discovery was completely neglected for a period of at least five decades. Only in the last two decades had the global health agenda seen a resurgence in research all around the globe that has led to the discovery of new molecules with anti-TB potential. Several new entities are being currently evaluated both in pre-clinical and clinical stages of drug development [2]. Despite that, only three new medications made it to the market: bedaquiline and pretomanid approved by the Food and Drug Administration (FDA) in 2012 and 2019, respectively, and delamanid, approved by the European Medicines Agency (EMA) in 2014. Very few molecules make it through the stringent bottlenecks of TB drug discovery because finding a new anti-TB drug is challenging: new compounds should kill *Mtb* with novel mechanisms of action showing rapid bactericidal activity, as well as activity against bacteria in different metabolic states without host toxicity. Even though the advances in understanding the biology of *Mtb,* including its complete genome sequence, have provided a platform of a wide range of novel drug targets, most of the compounds discovered in the last few years repeatedly target the cell wall (MmpL3 [3,4,5,6], DprE1 [7,8,9], FadD32 [10], and Pks13 [11]), while most of the approximately 625 essential *Mtb* genes are unexploited. With the approval of bedaquiline, which targets mycobacterial energy production [12], and delamanid, which targets both cell wall synthesis and energy production, the energy-metabolism in *Mtb* [12] has received significant attention in the last decade as a potential target to investigate and develop new antimycobacterial drugs.

In this review, we begin by providing a brief overview of *Mtb* energy-metabolism pathways. Next, we describe potent anti-mycobacterial agents targeting the energy-metabolism at different steps, with a special focus on structure-activity relationship (SAR) studies of the most advanced compound classes.

## 2. Energy-Metabolism in Mycobacterium Tuberculosis

It is well accepted that the inflow of carbon sources and maintenance of the proton motive force (PMF) that produce ATP are vital for microorganisms to grow, persist, and replicate in mammalian cells. In *Mtb*, such survival mechanisms are crucial for adapting to unfavorable environmental conditions such as hypoxia, unbalanced pH, and nutrient starvation [13]. Consequently, *Mtb* enters an anaerobic non-replicating and drug-tolerant state that, according to the WHO reports [1], represents a major concern for TB therapy. Latent *Mtb* (LTB) consumes the least energy for survival by cutting off many biosynthetic activities; in line with this, drugs targeting biosynthetic pathways become less efficient, leading to a widespread interest in exploiting the energy-metabolism of *Mtb* as a target [14].

Mycobacteria generate ATP via two inter-linked metabolic pathways: oxidative phosphorylation and carbon central metabolism (CCM). 

**Figure 1:**
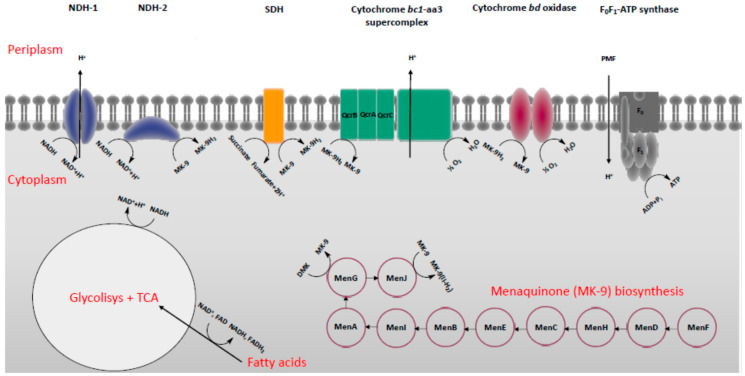
Schematic depiction of the energy metabolism pathways in *Mtb*.

In oxidative phosphorylation, the enzymes involved in the electron transport chain (ETC) create the electrochemical gradient PMF across the biomembrane, allowing the final step to occur: production of ATP [15]. Initially, electrons derived from the CCM enter the ETC through NADH dehydrogenase type I (NDH-1) and type II (NDH-2) or succinate dehydrogenase (SDH), which is also an integral part of the tricarboxylic acid (TCA) cycle [16]. These enzymes catalyze the oxidation of NADH to NAD^+^ and succinate to fumarate, respectively, followed by the menaquinone/menaquinol reduction. The menaquinone/menaquinol redox pool then transfers the electrons to a final acceptor (oxygen in aerobic conditions and nitrate or fumarate in anaerobic conditions) via terminal respiratory oxidases, while protons are pumped across the membrane, generating the PMF [17,18,19]. Despite an emerging role of fumarate reductase and nitrate reductase under hypoxia, in such conditions, the redox mediated by these enzymes is poorly understood, and a major focus is still given to the cytochrome bc1-aa3 “supercomplex” and the cytochrome bd oxidase, acknowledged to play a vital role in aerobic conditions. Eventually, energy incorporated in the PMF is exploited by the ATP synthase to produce ATP [20]. In line with this, targeting ETC enzymes together with those entailed in the menaquinone biosynthesis holds promise to provide the next generation of front-line drugs against TB (Figure 1) [21]. 

The CCM consists of an enzymatic transformation of carbon in order to provide energy in the form of reducing equivalents (NADH-2, FADH), ATP, and essential biosynthetic precursors for the growth and survival of the cell. A growing body of literature indicates that under anaerobic conditions, the metabolism of host fatty acids during the infection of *Mtb* is a core source of carbon substrate, discounting a role for carbohydrates. Therefore, in this condition, *Mtb* adopts β-oxidation, the glyoxylate shunt, the methylcitrate cycle, and cholesterol catabolism as main pathways coupled to TCA cycle to recycle carbon (Figure 1). Particularly, the glyoxylate shunt and the cholesterol catabolism have been gaining much attention owing to the lack of human homologues of most of their enzymatic targets. The glyoxylate shunt is a shortcut adopted by bacteria to skip the steps of carbon loss by CO_2_ formation in the TCA cycle. It uses isocitrate lyase (ICL) to cleave isocitrate into glyoxylate, which in turn is converted into malate by malate synthase to start another TCA cycle. Remarkably, many in vivo studies suggest the pivotal role of ICL during hypoxia induced by host macrophages. Furthermore, the role of host cholesterol in *Mtb* infection has been widely investigated, and besides the importance of its catabolism, cholesterol makes a potentially important contribution to the pathogenicity of *Mtb* by facilitating the entry of the mycobacteria into macrophages. In this context, oxidative phosphorylation, alongside with the glyoxylate shunt and cholesterol catabolism, emerge as distinctive pathways in carbon metabolism and represent a new target space for drug development in TB, and thus they deserve to be well-documented [21].

## 3. Classification of Drugs Targeting Energy-Metabolism in *Mtb*

### 3.1. Inhibitors of NDH-2

There are two types of NADH dehydrogenase in *Mtb:* (i) the multi-subunit-proton-pumping type I (NDH-1) and (ii) a single peptide chain non-proton-pumping type II (NDH-2) [22]. Although NDH-1 acts by transferring electrons to menaquinone to generate PMF, it was found to be not essential for *Mtb,* and thus, it has not been considered a promising target for drug discovery [16]. 

NDH-2 is a flavoenzyme characterized by two distinct binding sites that allow the enzyme to catalyze the transfer of electrons from NADH to menaquinone, likely by a two-site ping-pong or ternary kinetic mechanism [23]. Unlike NDH-1, NDH-2 is not found in mammalian mitochondria, and targeting NDH-2 has been indicated to be a valid anti-mycobacterial drug discovery strategy. [16,22]. 

Although further investigation on the ligand-binding pocket of the NDH-2 protein is necessary for structure-guided inhibitor design, numerous compounds inhibiting NDH-2 have been reported. 

#### 3.1.1. Riminophenazines

**Figure 2:**
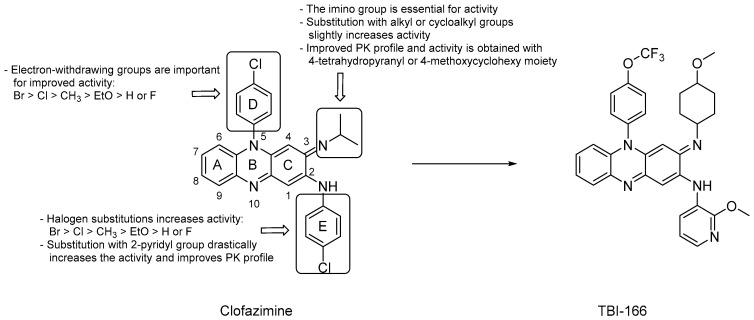
SARs of riminophenazines and chemical structures of clofazimine and TBI-166.

Riminophenazine derivatives are surely among the most commonly studied NDH-2 inhibitors. Clofazimine (CFZ, Figure 2), the lead compound of this class, showed a minimum inhibitory concentration (MIC) on 80 clinical *Mtb* isolates from patients with MDR-TB ranging from 0.079 to 2.53 μM for 75 of them and >2.53 μM for the rest. This suggests that CFZ may serve as a potential candidate for treating MDR-TB but, at the same time, new drug-resistant strains are developing [24]. The use of CFZ in the treatment of TB was abandoned due the unclear efficacy in in vivo models, unusual pharmacokinetic (PK) properties, and uncertain mechanism of action (MOA). However, according to WHO reports [1], it has been repurposed as part of treatment regimens for MDR-TB in Phase III trials. CFZ is a fat-soluble drug due to its high lipophilicity (log P = 5.39) [25] that, together with its long half-life (over 70 days in humans), [26] hampers its use in therapy. Furthermore, the long-term use of CFZ at the usual dose of 100 mg/day in patients with MDR-TB is associated with skin discoloration owing to tissue accumulation [27].

It is believed that CFZ acts as a prodrug that competes with menaquinone to be reduced by NDH-2 to release reactive oxygen species (ROS) upon re-oxidation by O_2_. In contrast, further studies suggest that the drug might exert a selective effect on DNA, and others speculate that the interaction with bacterial phospholipase A2 would turn toxic for mycobacteria [28].

Preliminary studies identified CFZ’s pharmacophore composed of a phenazine central core in resonance with two other aromatic rings at C2 and N5. The redox activity has been attributed to the nitrogen atoms in the phenazine structure and the R-iminium group at position 3. Many manipulations of the aromatic moiety of CFZ have been conducted, aimed at increasing both the bactericidal activity and the PK profile, and SARs can be summarized as following (Figure 2):

- deletion of the A, D or E aromatic rings led to a significant loss of the mycobactericidal activity [29];
- substitution of the iminium group at C3 with an alkyl or cycloalkyl group slightly increased the activity;
- halogen substitution on the phenyl rings was not essential for the anti-TB activity, but when substituted at the para-positions, activity increased according to the following order: Br > Cl > CH_3_ > EtO > H or F;
- both the phenyl rings at C2 and N5 can be substituted with a pyridyl ring and, notably, the replacement with a 2-pyridyl group at C2 drastically increased the activity and led to favorable PK properties [29];
- replacing the isopropyl group on the imino nitrogen with either a 4-thetrahydopyranyl or a 4-methoxycyclohexyl moiety led to improved activity and more favorable PK [30];
- electron-withdrawing groups decorating the N5 phenyl ring play a considerable role in retaining the activity, and the halogen substitution pattern seems to be important for cytotoxicity [30,31].

The TB Alliance in collaboration with the Institute of Materia Medica, the Chinese Academy of Medical Sciences, and the Peking Union Medical College in Beijing, in a project aimed at optimizing CFZ, found TBI-166 (Figure 2), a riminophenazine derivative endowed with promising activity. Compared to CFZ, in vivo studies demonstrate that TBI-166 has an equivalent efficacy at the same dose, but showed a shorter half-life (41.25 h) and a decreased lipophilicity (log P = 4.52), likely due to the replacement of the C2 phenyl ring of CFZ with 2-methoxypyridyl group. Taken together, these properties lead to a reduction of skin discoloration and better PK parameters (increased Cmax and AUC) with respect to CFZ [32,33]. TBI-166 completed preclinical development in 2015 and in January 2018 was selected as a candidate for a phase I clinical trial. 

#### 3.1.2. Quinoline/Quinolone Derivatives

Several studies have speculated different biological activities of quinoline and quinolones against bacteria, tumors, and malaria [34]. A growing body of literature is putting a focus on the anti-TB properties of such derivatives [35], and currently, the quinoline-based derivatives moxifloxacin and bedaquiline are being studied in clinical trials to be used in new drug regimens for the treatment of both drug-susceptible and drug-resistant TB. Furthermore, some *Mtb* isolates expressing resistance to first-line therapy are susceptible to fluoroquinolones. Therefore, they are used as a second-line treatment for MDR-TB [1].

**Figure 3:**
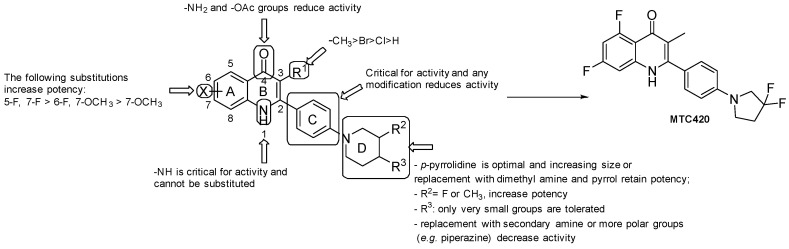
SARs of Quinoline/quinolone derivatives and the chemical structure of MTC420.

Recently, several quinoline or quinolone-based derivatives have been identified as NDH-2 inhibitors. From a high-throughput screening (HTS) of 11,000 compounds predicted to have an inhibitory activity towards NDH-2, Hong et al. identified some promising quinolone candidates, and the pharmacophore depicted in Figure 3 was investigated for optimization, and SARs were carried out (Figure 3) [36]:

- the presence of -NH2 and -OAc at the 4-position led to inactive molecules;
- the replacement of the phenyl ring at position 2 with a pyridyl ring or any other modification resulted in a loss of activity;
- the most favorable X groups on the A ring are: 5-F, 7-F > 6-F, 7-OCH_3_ > 7-OCH_3_;
- any substitution of the H on the N1 nitrogen led to a loss of activity;
- substitution at C3 with H and halogenation led to a loss of activity. Compound-specific exception resulted from substitution with Br, but generally, the -CH_3_ group is considered optimal at this position;
- the piperidine of the side chain has to be located at the *para* position; small groups at the 4-position are more tolerated than larger ones, such as -CF_3_ and cyclopropyl; the F and -CH_3_ groups at the 3-position resulted in improvements in anti-TB activity; when increasing the ring size of the piperidine, the presence of dimethyl amine and substitution with a pyrrole retained potency.

MTC420 (Figure 3) was selected as the lead compound. Indeed, MTC420 displayed acceptable anti-TB activity (*Mtb* IC50 = 525 nM, *Mtb* Wayne IC50 = 76 nM, and MDR *Mtb* patient isolates (IC50 = 140 nM) with favorable PK and toxicological profiles. In vivo efficacy studies for this compound are currently under evaluation.

**Figure 4:**
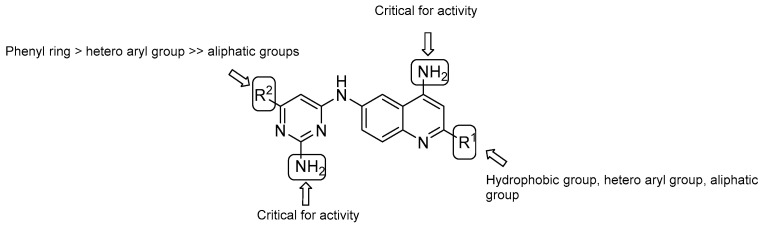
SARs of quinolinyl pyrimidine derivatives.

AstraZeneca Corporate identified quinolinyl pyrimidines as one of the most promising classes of NDH-2 inhibitors by using an HTS format of 100,000 compounds. Further studies revealed that a quinolinyl pyrimidine with a scaffold of two primary amines (Figure 4) is pivotal for the activity and may undertake critical hydrogen-bonding interactions with the NDH-2 target protein. Therefore, efforts to understand the SARs of this scaffold and improve the biochemical potency against the target enzyme were performed (Figure 4) [37]:

- both –NH_2_ groups on the quinolone and the pyrimidine rings are critical for activity;
- in vitro evaluation showed an increasing potency regarding the monosubstitution at R^2^ in the following order: phenyl ring > hetero aryl groups >> aliphatic groups;
- on the quinoline ring, substitution at R^1^ displays a similar trend as the pyrimidine ring (R^2^), with the most potent substitution group to be a 4-F or 2-OCH_3_-phenyl ring.

Data suggested that these compounds undertake hydrophobic interactions with NHD-2. The representative compounds of this class showed low solubility parameters (logD = 2.16-3.18) and MICs ranging from <0.81 to 2.88 μM, making quinolinyl pyrimidines an interesting scaffold for further optimization.

**Figure 5:**
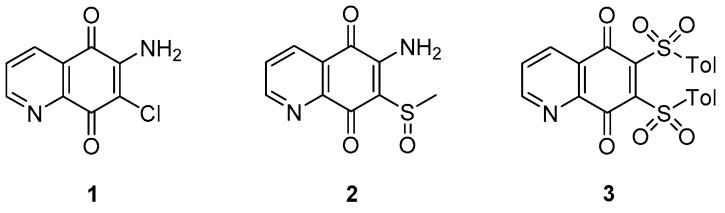
Chemical structures of compounds 1–3.

Finally, a study of 6,7-substituted-5,8-quinolinequinones (QQs) afforded compounds with specific anti-TB activities (MICs ranging from approximately 0.81 to 2.88 μM) that needed further investigation [38]. Generally, compounds exhibited unclear TB activity and a precise SAR could not be made. However, analysis at both the 6 and 7 positions suggested that halogens are favorable substituents compared to thiol groups and compounds with a free amine group at position 6 may exhibit a relevant anti-TB activity according to the nature of the substituent at position 7. Three quinoline derivatives (1, 2 and 3, Figure 5) with micromolar MICs (6.60–28.7 μM) were chosen as candidates for further analysis and structure optimization. 

#### 3.1.3. Thioquinazolines and Tetrahydroindazoles

**Figure 6:**
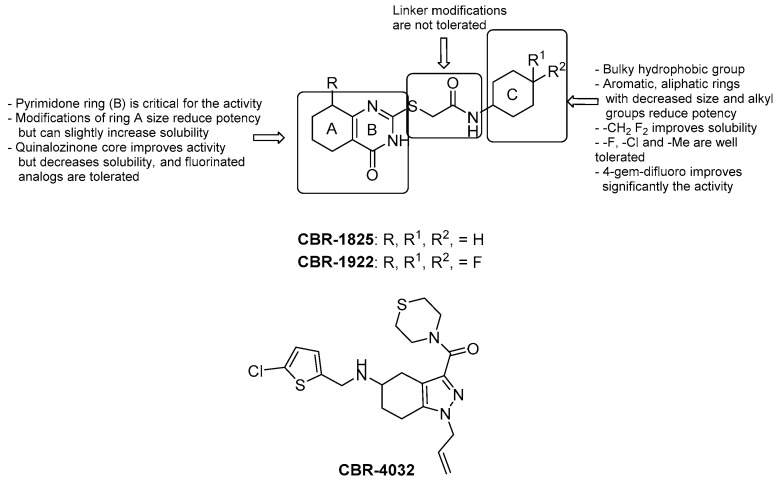
SARs of TQZ and THI and chemical structures of CBR-1825, CB-1922, and CBR-4032.

HTS of over 800,000 compounds was performed by utilizing membrane vesicles derived from *Mtb* upon the addition of NADH. The thioquinazoline (TQZ, Figure 6) CBR-1825 and the tetrahydroindazole (THI, Figure 6) CBR-4032 blocked NDH-2 and not SDH; furthermore, their MIC_50_ values against *Mtb* were 0.43 μM and 6.6 μM, respectively, while they proved to be safe for mammalian cells [39]. Murugesan et al. reported additional experiments on TQZ, including PK studies [40]. Altogether, SARs of TQZ can be summarized as follows (Figure 6):

- the pyrimidone core (unit B) cannot be modified;
- generally, increasing/decreasing the saturated ring size brings a reduction of activity, although the solubility slightly increases with the related 5-membered ring;
- replacement of the saturated fused ring with a phenyl (quinazolinone) core increases the anti-TB activity; fluorine analogues were synthesized to increase stability but are less soluble;
- oxidation or replacement of the thioether with O, N, Me and N-methylation of the amide and/or the methylene linker induces to a reduction of activity; thus, the linker modification is generally not tolerated;
- contraction of the cyclohexyl amide at the side chain to cyclopropane or cyclobutane leads to a decrease of activity that is almost retained with cyclopentane and cycloheptane; furthermore, substitution with aromatic rings or disruption of the cycle increases MIC values, suggesting that a bulky hydrophobic group is required to obtain good whole-cell potency;
-F, -Cl, and -Me substituents are well tolerated on the cyclohexyl ring, with the best activity reached upon substitution with a 4-gem-difluoro;
- solubility significantly increases with -CH_2_-gem-difluoro cyclohexyl at the end of the side chain.

Accordingly, the introduction of 4-gem-difluoro on the cyclohexane and of the 5-F-quinazolinone core yielded the discovery of CBR-1922 (Figure 6) as the utmost potent TQZ (MIC = 0.09 μM). PK studies suggested that the in vitro oxidation of the cyclohexyl and quinazolinone ring, the cleavage of the amide and, more importantly, the reactivity of the linker with the glutathione might reduce in vivo activity [40].

Several data suggest that the TQZ and THI series target NDH-2 [39]:

- resistant *MTb* strains showed 50-fold higher expression of the gene *ndhA;*
- neither compound (100 μM) inhibited the growth of *M. smegmatis* at 48 °C when NDH-2 activity is replaced by the malate/menaquinone oxidoreductase;
- TQZ and THI scaffolds are related to the quinone and adenine molecules;
- TQZ has been previously reported to inhibit the NDH-2 [41].

#### 3.1.4. Iodonium Derivatives

**Figure 7:**
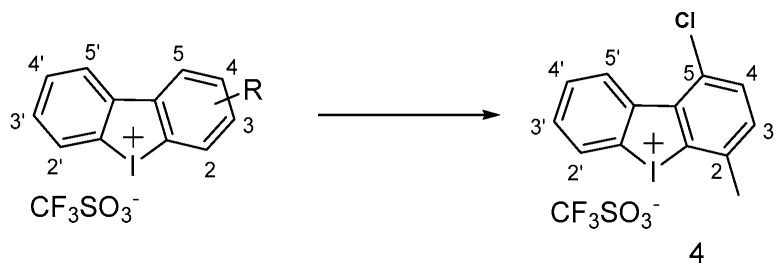
Chemical structures of DPI and compound 4.

**Figure 8:**
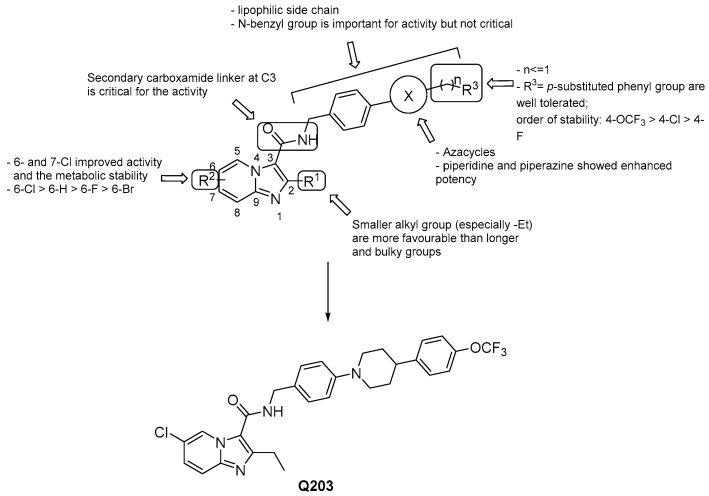
SARs of imidazo [1,2-*a*] pyridine-3-carboxamides and chemical structure of Q203.

A few early reports led to the discovery of iodonium compounds as potential inhibitors of NDH-2 [42,43]. Diphenyliodonium (DPI, Figure 7) and its analogues were shown to inhibit *Mtb* at micromolar concentrations [44]. A systematic SAR study on a set of DPI analogues was conducted for the first time by Nguyen et al. in 2018, who identified a series of compounds with significant activities against *Mtb* (MIC = 0.13-4.4 μM). Briefly, the first series of DPI displayed a significant improvement of in vitro activity upon halogen substitution at position 5, and Cl proved to be the best one. The second DPI series focused on analogues with a halogen at the 5-position and, notably, the analogue with 5-Cl and 2-Me was three-fold more potent than the DPI itself (MIC = 0.13 μM) (compound 4, Figure 8) [45]. Notwithstanding, the potent antimicrobial activity of DPI has always been hampered by its corresponding toxicity to mammalian cells, and the current challenge for DPI is its narrow therapeutic index (TI). Hence, the same authors assessed the cytotoxicity against HEPG2 cells, showing that improved pathogenicity is not a prerogative for higher toxicity but, unfortunately, the best candidates selected for their potency (DPI 5-substituted) are still rather toxic and need to be further optimized.

### 3.2. Inhibitors of Cytochrome bc1

To date, the resolution of the atomic structure of the cytochrome bc1-aa3 “supercomplex” is available: the cytochrome bc1-aa3 “supercomplex” consists of two transmembrane enzymes tightly associated, the cytochrome bc1 complex and cytochrome aa3 oxidase, which are structurally and functionally related to the mitochondrial complexes III and IV, respectively. Basically, in contrast to mitochondria, the cytochrome bc1 transfers electrons from the menaquinol to the cytochrome aa3 oxidase via the QcrC domain, instead of using a soluble cytochrome c as a shuttle. Consequently, the cytochrome aa3 oxidase pumps protons across the membrane [46]. This pathway is the most energetically favorable during the respiratory process. Nonetheless, the genetic knockout of the cytochrome bc1-aa3 “supercomplex” only decreased growth both in vitro and in vivo [47], suggesting that the bacterium attempts to maintain a favorable membrane potential for ATP production through an increased cytochrome bd activity.

Following the discovery of imidazopyridine as cytochrome bc1-aa3 inhibitors, several classes of compounds have been developed for the same target, and herein we describe those that have generated considerable interest and whose SAR have been reported. Interestingly, all these inhibitors appear to act by binding the b subunit of the cytochrome bc1 complex (QcrB). Few researchers have addressed the problem that current work focuses only on a specific part of the supercomplex and it may increase the odds for drug-resistance at that level. Therefore, this observation stresses the importance to understand in more detail the respiratory oxidases mechanism and to conduct a rational design of small molecules targeting a novel site within the supercomplex.

#### 3.2.1. Imidazo [1,2–a] pyridine-3-carboxamides

Imidazopyridines have been identified as inhibitors of the energy production in *Mtb* by several groups [48,49]. In particular, the imidazo [1,2-a] pyridine amides (IPAs) class is surprisingly highly selective to mycobacteria [48,50], and the most advanced derivatives showed MICs in the nanomolar range. The discovery of Q203 (Telacebec, Figure 8) [51], the most advanced compound of this class, has prompted many to lead optimization programs around the IPA scaffold providing elaborated SARs:

- 6- or 7-Cl groups at the R^2^ position enhance both the activity and the metabolic stability with respect to the unsubstituted compounds [52,53,54];
- the ethyl group at R^1^ appears to be the most favorable [52,53,54];
- a lipophilic side chain is pivotal for the activity, regardless of chain length and linearity [52];
- the substituted *N*-benzyl group at the side chain is important but not critical for the activity [53,54,55,56,57];
- 4-trifluoromethoxy-phenyl-piperidino group is the best one at the side chain, but can be replaced by other nitrogen heterocycles to improve PK properties [55,58];
- generally, scaffold switching is not a good option since that of the imidazo [1,2-a] pyridine-3-carboxamides is optimal for potency and ADME properties [59], but there are a few exceptions [60,61,62];
- the presence of -NH in the carboxamide group at 3 is critical for the activity, as well as the carbonyl group [53,54];
- switching the position of the carboxamide from 3 to 2 results in less effective derivatives.

The binding mode of IPAs to QcrB has been recently validated by using in-cell NMR [63]. In this work, the binding of the Q203 derivative, namely IPA317, was shown to interact directly with the residue T313, which was previously identified as a potential position conferring resistance to IPAs [50,51]. Recently, molecular hybridization has gained importance to elude the drug-resistance mechanism. In light of this, a few works designed new sets of hybrids with imidazo [1,2-a] pyridine as an active pharmacophore [57], but they did not afford highly active compounds. Therefore, it remains a challenging area to explore.

Q203 remains the best candidate of the IPAs family. Indeed, it showed activity in the nanomolar range against *Mtb* (MIC_50_ = 2.7 nM) that was conserved for MDR and XDR clinical isolates; likewise, a safety profile of the drug was recorded since it did not inhibit either CYP450 targets or the hERG (human ether-à-go-go related gene) potassium channel. In addition, despite the high lipophilicity (LogP: 7.64) [56], Q203 was well-tolerated during acute and long-term administration in mice, and the PK profile (low volume of distribution with a drug concentration in lungs 2-3-fold higher than in serum, bioavailability of 90% and a terminal half-life of 23.4 h) was even more reassuring than in BDQ [52]. Pre-clinical studies confirmed the high efficacy of Q203 as an anti-TB drug, and Phase 1 clinical study showed it is well-tolerated in healthy human subjects. In June 2019, Q203 passed the Phase 2a EBA (Early Bactericidal Activity) clinical trial (NCT03563599), showing promise for its use in therapy alone or in combination and bringing high hopes for the development of new optimized IPAs as a class of medicine. Currently, it is being evaluated for safety, tolerability, and pharmacokinetics [64].

#### 3.2.2. 2-(Quinolin-4-yloxy) Acetamides

**Figure 9:**
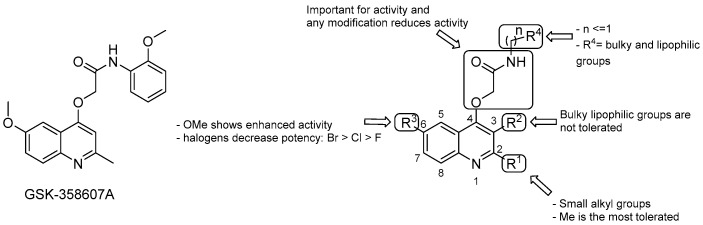
SARs of 2-(quinolin-4-yloxy) acetamides and the chemical structure of GSK-358607A.

The 2-(Quinolin-4-yloxy) acetamides (QOAs) are non-cytotoxic compounds endowed with micromolar MICs against *Mtb,* identified from a phenotypic screening campaign carried out by GSK and others [65]. Various efforts to explore the SAR of this series have been reported based on the hit compound GSK-358607A (MIC 0.70 μM, Figure 9) [65,66,67,68]:

- the Me group at R^1^ seems to be the most suitable one, and as alkyl chain length increases, the potency diminishes;
- the presence of a bulky lipophilic benzyl group at R^2^ results in a decrease of potency for the respective compound;
- the best group at R^3^ is the methoxyl one; replacement with halogens decreases the potency (Br > Cl > F);
- bulky and lipophilic substituents of limited conformational flexibility (*n* ≤ 1) at R^4^ improve anti-TB activity, regardless of the aromaticity and planarity of the eventual ring;
- the oxygen at position 4 plays an essential role for the activity; thus, substitution with NH to increase the solubility is not tolerated;
- removal or switching to a secondary group or even replacement with bioisosters [69] of the primary amide at the side chain decreases efficacy.

Although SARs may suggest a correspondence between lipophilicity and potency of this series, the lipophilicity alone cannot explain the improved anti-mycobacterial activity. For instance, compounds with similar CLogP displayed different MIC values [68]. Notably, in the same study, QOA presenting a pentyl substituent at the *p*-position of the phenyl ring of the side chain (Figure 9) displayed a MIC as low as 0.005 μM that, to our knowledge, is the lowest value reached so far for this series. This compound series showed mild cardiotoxicity and low toxicity toward mammalian cells as well [66,68]. Nevertheless, they also showed moderate metabolic stability, both in mouse plasma and mouse liver microsomes, attributed in part to the amide group lability [69]. In an attempt to improve the metabolic profile, recently, Borsoi et al., via molecular simplification of the attached acetamide group, provided molecules that were metabolically more stable than their counterparts, which retained submicromolar activity against MDR-*Mtb* strains [70]. 

Although it was demonstrated that QOAs targets Qcrb by both whole-gene sequencing of resistant mutants and gene deletion studies, there is still uncertainty regarding whether QcrB is the only physiologically relevant target [66,71].

#### 3.2.3. New Chemical Entities

More recently, novel validated QcrB inhibitors chemically distinct from previously reported compounds have been identified. 

**Figure 10:**
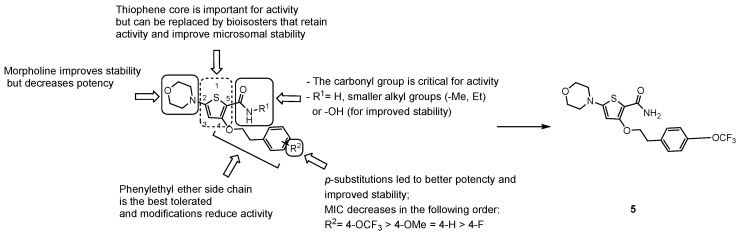
SARs of morpholino-thiophenes and the chemical structure of compound 5.

From a cell-based phenotypic screening against *Mtb*, a class of novel morpholino-thiophenes (MOT) analogues was identified [72]. The hit compound of this series displayed submicromolar activity (MIC_90_ = 0.72 ± 0.30 μM) and good PK and toxicity profiles, although low metabolic stability in mouse microsomes was detected. From an initial SAR investigation of the pharmacophore depicted in Figure 10, it emerged that [72]:

- the carbonyl group of the primary amide is fundamental for the activity and only substitution of the primary amide, to retain activity (e.g., *N*-methyl-amide) or to increase metabolic stability (e.g., hydroxamic acid), are tolerated;
- the morpholine ring cannot be replaced. Only a few modifications to improve metabolic stability are allowed (e.g., morpholine-3-one), but decrease potency;
- only an ethyl ether linker attached to a *p*-substituted phenyl ring is tolerated at position 4; moreover, constraining the ethyl linker led to a loss of activity even if it improved stability, and saturation or heterocyclic substitution of the phenyl ring increases polarity but induces a loss of potency;
- the thiophene core may be replaced by bioisosters to improve microsomal stability while maintaining whole-cell activity.

Accordingly, structure optimization of the hit compound led to the discovery of the lead compound 5 (MIC_90_ = 0.24 ± 0.0098 μM, Figure 10), which displayed improved microsomal stability, although poor aqueous solubility (1 μg/mL), low oral bioavailability (12–18%), and modest in vivo activity in an acute infection TB-mouse model are drawbacks that still need to be improved.

**Figure 11:**
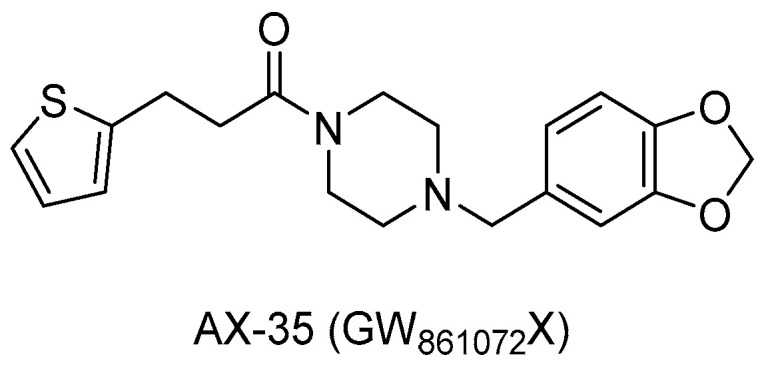
The chemical structure of AX-35.

From a large screening campaign led by GSK, 177 hit compounds were found to be active against mycobacteria [73]. Among these, a more recent work evaluating the anti-TB activity of AX-35 (GW861072X, Figure 11), an arylvinylpiperazine amide, and analogues could be designed to assess the importance of the sulfur atom in the thiophene ring for antimycobacterial activity. Nevertheless, AX-35 was the most active and most selective compound within this series (MIC = 0.14 µM). All the tested compounds presented low or no toxicity, but low metabolic stability. Different positions of S on the thiophene ring or its replacement with bioisosters, such as phenyl and thiazole, did not ameliorate the drug profile, and in the future, optimization to enhance the potency and stability of this series is needed [74].

**Figure 12:**
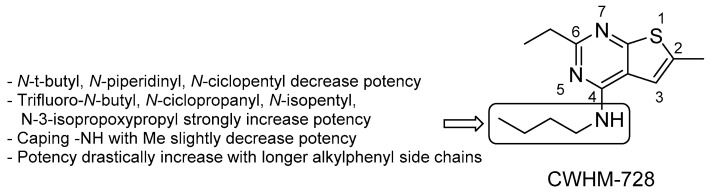
SARs of 4-amino-thieno[2,3-*d*]-pyrimidines and the chemical structure of CWHM-728.

Another screening of 78 small-molecule nucleotide mimetics [75] identified 4-amino-thieno[2,3-*d*]-pyrimidines which inhibit the growth of *M. smegmatis.* The improved analogue CWHM-728 (Figure 12) inhibits *Mtb* growth with an IC_50_ of 2.7 ± 0.84 μM [75], and it was then selected as the lead compound for initial SAR studies focusing on the side chain. The introduction of a 3-phenylpropyl side chain led to an increase of 38-fold in potency versus *Mtb* (IC_50_ = 83 ± 5.4 nM). Accordingly, 4-amino-thieno[2,3-*d*]-pyrimidine looks like a promising scaffold for further derivatization. 

**Figure 13:**
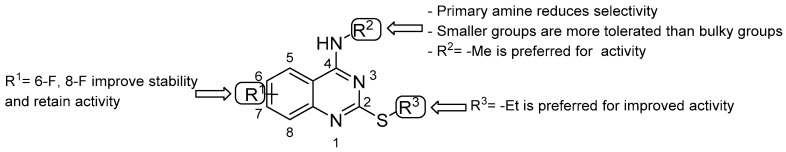
SARs of quinazolines.

Finally, a new series of quinazoline derivatives (2-ethylthio-4-methylaminoquinazoline) with considerable activity against *Mtb* was discovered, and preliminary SAR can be summarized as follows (Figure 13) [76]:

- double substitution with two F atoms at both positions 6 and 8 improves the stability of the metabolism;
- the ethyl group at the thioalkyl side chain (position 2) is the most suitable;
- the secondary amine at position 4 with the smallest substitution (methylamine) is more effective for anti-TB activity; the primary amine or bulky substituents have a detrimental effect on the activity.

#### 3.2.4. Repurposed Drugs: Zolpidem and Lansoprazole

**Figure 14:**
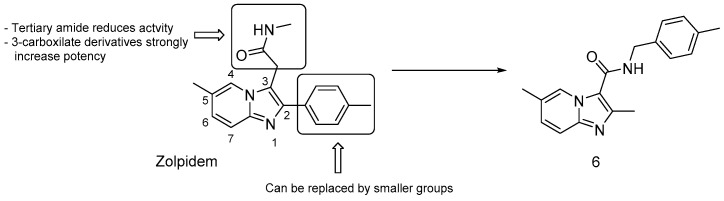
The chemical structure of Zolpidem and SARs of its analogues.

In an effort to repurpose drugs for anti-TB therapy, Zolpidem (Figure 14), known for its sedative effect, showed moderate inhibitory activity against *Mtb* (MIC = 10−50 μM). Zolpidem proved to inhibit the cytochrome *bc1* complex at the QcrB level along with imidazo-[1,2-a]-pyridine-3- carboxamide antitubercular agents, which bear similar structure, and thus, similar SAR [59]. To enhance its potency, manipulations around the Zolpidem scaffold were carried out, and anti-TB assays revealed that (Figure 14) [59]:

- bulky groups at position 2 are not essential; thus, the 2-tolyl moiety in Zolpidem can be replaced by a methyl group;
- 3-carboxilate derivatives are much more potent than 3-oxoacetamide and 3-acetamide derivatives;
- secondary amides are more effective than tertiary amides that lack a hydrogen bond donor.

Accordingly, the 3-carboxilate derivative bearing a 4-methyl-benzyl amino group (compound 6, Figure 14) was the most potent analogue with a MIC of 0.004 μM, highlighting the potential of the imidazo-[1,2-*a*]-pyridine-3-carboxamide scaffold in TB therapy. Recently, more Zolpidem structural modifications led to the discovery of imidazo-[1,2-a]-pyridine/pyrimidine-1,2,3-triazoles (IPTs), which exhibited a two-fold higher potency against *Mtb*, and might represent new leads for developing potent anti-TB drugs [77].

**Figure 15:**
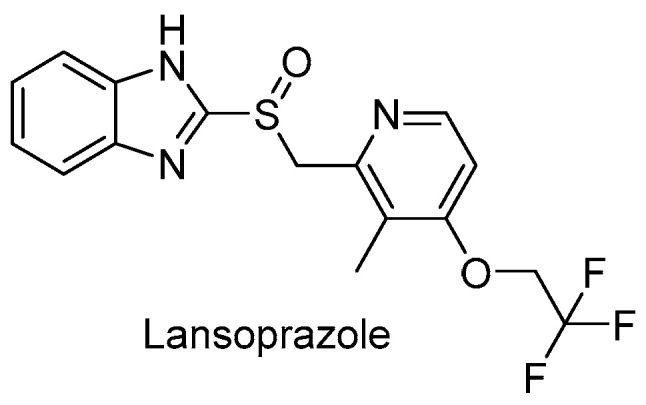
The chemical structure of lansoprazole.

Lansoprazole (LPZ, Figure 15), a gastric proton pump inhibitor, was also identified by a pharmaceutical repurposing screening of 1280 FDA-approved drugs and is active against *Mtb* residing within MRC-5 lung fibroblasts cells with an IC_50_ of 1.47 μM. Interestingly, LPZ’s ex vivo activity is 22-fold higher than its in vitro activity, suggesting that LPZ is a prodrug that undergoes rapid conversion to an active form within the host cytoplasm. Then, lansoprazole sulfide (LPZS) was established as the active compound that showed high selectivity towards *Mtb,* and no interaction with gastric H^+^K^+^-ATPase. This derivative likely targets QcrB via a unique binding mechanism, as T313A Qcrb mutants are still susceptible to its action [78]. However, oral administration in rats is not sufficient to reach a therapeutic effect in the lungs [79]. Therefore, other routes of administration should be considered while future SAR studies are in progress. 

### 3.3. Inhibitors of Cytochrome bd

Although the cytochrome *bc*_1_ is proposed to be bioenergetically more efficient than cytochrome *bd* oxidase, *Mtb* is able to adapt and survive during the inhibition of cytochrome *bc*_1_ by inducing overexpression of cytochrome *bd* [80]. Therefore, QcrB inhibitors lack early bactericidal activity and are meanly bacteriostatic. In light of this, the role of cytochrome *bd* has been reevaluated, and researchers are taking into account the idea of targeting both cytochrome *bc*_1_ and cytochrome *bd* by using a combination of antibiotics. Although this approach is interesting, it suffers from a lack of validated inhibitors of cytochrome *bd.* To date, aurachin D remains the only selective cytochrome *bd* inhibitor [81,82,83], and the concurrent therapy with Q203 was demonstrated to have a synergistic bactericidal effect against *Mtb* [84]. Hence, cytochrome *bd* oxidase offers an intriguing area to develop new TB drugs with potential use in combination therapy. 

### 3.4. Inhibitors of Menaquinone Biosynthesis

It is well known that menaquinone (vitamin K2) plays an important role in producing the electrochemical gradient required for the production of ATP in ETC by shuttling electrons to terminal reductases. Therefore, each step involved in menaquinone biosynthesis, catalyzed by Men enzymes (MenA-J), is a potential chemotherapeutic target; besides, unlike bacteria, human cells are unable to produce menaquinone *ex novo*, as it is obtained only through the diet [17,18,19]. The most promising results come from inhibitors of Men-enzymes that function downstream of the synthesis pathway (MenA, MenB, MenG), of which DG70 is the most recent compound.

#### DG70

**Figure 16:**
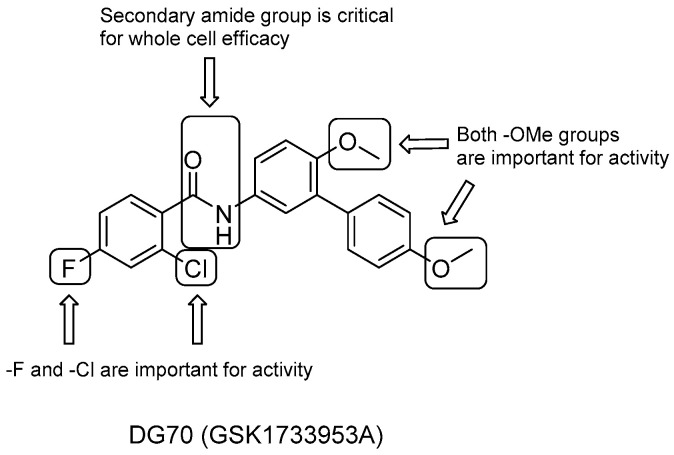
The chemical structure of DG70 and SARs of analogues.

A work involving a novel respiratory pathway-specific whole-cell screening of a library of 168 novel inhibitors of *Mtb* with unknown mechanisms of action carried out by Sukheja et al. [85] identified the biphenylbenzamide DG70 (GSK1733953A, Figure 16), which has a selective activity against both *Mtb* (MIC = 12.4 mM) and drug-resistant strains (MIC = 3.1–24.8 mM). Whole-genome sequencing of DG70-resistant colonies, radio-labeling, and high-resolution mass spectrometry studies identified DG70 as the first small-molecule inhibitor of MenG (demethylmenaquinone methyltransferase) [85]. The latter catalyzes the final step in the bacterial production of menaquinone through the methylation of demethylmenaquinone by using the cofactor S-adenosylmethionine (SAM).

A focused set of DG70 analogues was then prepared to carry out first SAR studies (Figure 16) [85]:

- the role of the two methoxyl groups appears to be critical for whole-cell efficacy since either removal or substitution with -OH diminishes the activity; a similar loss of anti-TB activity is observed with the eradication of the terminal methoxyphenyl moiety;
- removal of either the F or both F and Cl on the benzoyl moiety causes a small decrease in activity, which is completely lost after the elimination of the solely Cl group;
- the presence of secondary amide is also critical to whole-cell efficacy.

DG70 also showed low cytotoxicity in Vero cells (CC_50_ > 77μg/mL), and high bactericidal activity in vitro was obtained in combination therapy with other anti-TB drugs, such as INH and BDQ. On the other hand, modest aqueous solubility (0.66 μg/mL at pH 7.4) and poor stability in mouse liver microsomes stem the rout for in vivo efficacy assessments ([*t*_1/2_] = 0.753 min). Hence, optimization studies and consequent in vivo efficacy assessments are still required to offer a new component in TB therapy. 

### 3.5. Inhibitors of F_o_F_1_-ATP Synthase (ATPase)

ATP synthase consists of a membrane-embedded part (F_o_), which transfers protons from the periplasm to cytoplasm, and a hydrophilic part (F_1_), where ATP synthesis takes place. F_o_ comprises a rotary ring of nine c subunits, whereas the F_1_ complex is made up of α3β3γδε subunits [86,87]. The passage of H^+^ ions causes the rotor ring F_o_ to rotate, and the rotational motion is transmitted to the α and β catalytic core of unit F_1_ through the connecting subunits γ and ε, resulting in ATP synthesis [88]. In many bacteria, ATP synthase can undergo an opposite reaction to maintain the PMF under hypoxic conditions. A unique feature of *Mtb* ATP synthase is the suppression of the ATP hydrolase activity and its inability to establish a proton gradient. This could be an adaptive mechanism to prevent ATP waste under low oxygen conditions [89,90].

#### 3.5.1. Diarilquinolines (DARQs)

**Figure 17:**
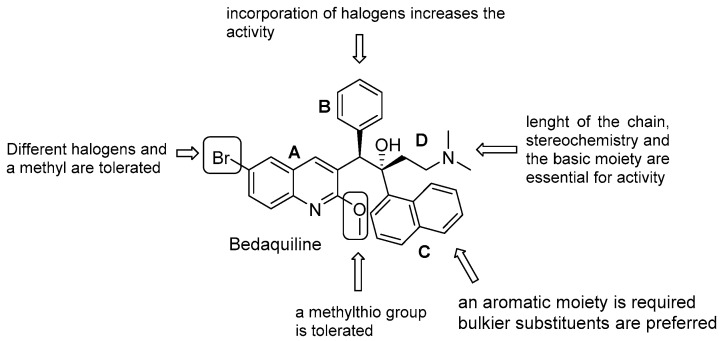
The chemical structure of bedaquiline and SARs of analogues.

The first-in-class diarylquinoline bedaquiline (BDQ, Sirturo^®^, Janssen-Cilag International NV, Beerse, Belgium, Figure 17) was approved by the FDA in 2012 and by the EMA in 2014 as an addition to treatment options for MDR-patients. It was identified through a whole-cell phenotypic screening and exhibited an outstanding bactericidal activity against replicating and dormant Mtb, no cross-resistance with other anti TB drugs, and a sterilizing activity in animal models [91]. Bedaquiline targets ATP-synthase, and a combination of extended biophysical, biochemical, and genetic studies show three possible mechanisms of actions. Two involve direct interaction with the enzyme and a dual binding both to the *c* subunit of the rotor F_o_, which causes stalling of the rotation of the *c* ring, and the ε subunit, which prevents the ability to connect rotation to ATP synthesis [92]. An additional mechanism is the uncoupling of the transmembrane proton flow and collapse of the PMF [93].

BDQ is a pure enantiomer and consists of a central quinoline nucleus decorated with a functionalized C3 side chain, which accounts for the difference from other quinolines and quinolone families [94]. An intense optimization process of the DARQ scaffold highlighted the main features for activity and 4 essential structural units (Figure 17):

- quinoline core (Unit A): (i) omitting substituents at C2 and C6 is deleterious for the activity; (ii) different halogens or a methyl moiety at position 6 are allowed, but an excess of electron density is not tolerated; (iii) a methylthio moiety can replace the methoxy at C2; (iv) the combination of a halogen at position 6 and methoxy or methylthio at position 2 gives the optimal activity;
- first phenyl ring (Unit B): incorporation of halogens increases the activity;
- second phenyl ring (Unit C): (i) a lipophilic group is essential for activity; (ii) an aromatic ring is required; (iii) an electron-withdrawing substituent and di-substituents on the phenyl ring are well tolerated; (iv) a bulkier substituent is preferred;
- C3 side chain (Unit D): this unit does not tolerate modifications. The two hydrogen bonding acceptors/donors are crucial for activity and any attempt at modifying the length of the chain, the basicity of the amino group, and the configuration of the two stereogenic centers was detrimental.

**Figure 18:**
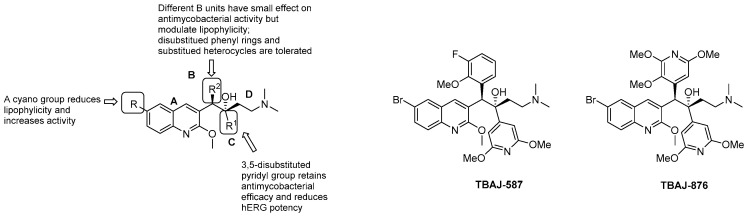
SAR for 2nd generation DARQs and chemical structures of the two pre-clinical candidates TBAJ-876 and TBAJ-587.

BDQ showed promising results in a clinical setting. In a Phase IIb for MDR-TB, its use in a five-drug regimen resulted in a faster sputum-culture conversion but was related to a higher risk of death compared to the control group [95]. However, trial deaths were not associated with its side-effects, and BDQ is currently undergoing multiple clinical trials in combination with other drugs, reducing overall mortality and improving treatment outcomes when added to MDR and XDR regimens [96,97]. The pharmacological and toxicological liabilities of BDQ are related to its high lipophilicity, which is likely to be associated with hepatotoxicity, tissue accumulation, and acquired resistance, due to its long half-life (up to 4–6 months) [12]. Even though information is limited and more robust data are needed to define its long-term use and cardiovascular safety profile, BDQ administration was associated with a prolonged QT interval, and the FDA included a black box warning of increased risk of QT prolongation and sudden death [98]. BDQ indeed inhibits hERG channels, a marker for potential arrhythmogenic risk and prolonged QT interval. The hERG potency is increased upon the basicity of the dimethylamino group, which is protonated at physiological pH. A critical factor for BDQ optimization is to then get a proper balance of the lipid solubility and basicity of the terminal dimethylamino group [99]. There is a great interest in manipulating the BDQ scaffold to obtain second-generation DARQ derivatives with attenuate hERG inhibition and reduced lipophilicity [100,101,102,103,104,105,106]. A TB Global Alliance collaborative project allowed the selection of two compounds (TBAJ-876 and TBA-587, Figure 18, Table 1). TBAJ-876 entered Phase 1 clinical trial on June 8, 2020. In this medicinal chemistry campaign, the SAR of diarylquinoline was systematically explored by replacing the 6-Br substituent with a more polar cyano group, the B-phenyl with heterocycles, and di-substituted phenyl rings and the C unit with either substituted pyridines or different heterocycles (Figure 18) [107,108,109,110,111,112].

Only the C-pyridyl analogs had a reduced affinity for hERG channels, along with retained antimycobacterial activities in vitro and in vivo, lower logP, and a predicted higher clearance in humans (Table 1).

TBAJ-876 shares BDQ’s ability to inhibit ATP synthase through the dual targeting of *c* and ε subunits [92] but does not retain the uncoupler activity [113]. This might be due to its reduced lipophilicity, which limits its capability to diffuse through the membrane. Interestingly, this does not affect the bactericidal activity, suggesting that uncoupler properties are not crucial for antimycobacterial activity [113]. Similarly to BDQ, TBAJ-876 proved to be active in vitro and in vivo against *M. abscessus* complex, representing a potential alternative also for *M. abscessus* lung disease [113].

#### 3.5.2. Squaramides

**Figure 19:**
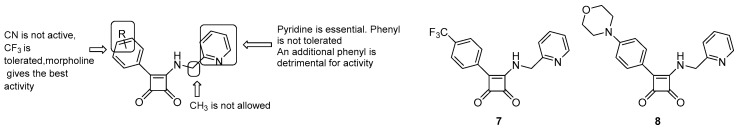
SAR analysis for the squaramide class and chemical structures of hit 7 and its improved analogue 8.

A HTS of more than 900k compounds from the Astra Zeneca collection used to identify disruptors of the ATP synthesis pathway led to the identification of the squaramide class [49]. The initial hit 7 (Figure 19) showed an acceptable enzymatic potency and in vitro antimycobacterial activity. Further medicinal efforts afforded an improved hit (Compound 8, Figure 19) for in vivo proof-of-principle studies and allowed SAR analysis (Figure 19), which can be summarized as follows:

- a 2-pyridyl moiety is critical for activity, suggesting that the nitrogen is involved in hydrogen bonding with the target;
- replacing the -CF_3_ group with a more hydrophylic one (such as a cyano) is detrimental for activity, whereas a morpholino ring, having the potential for hydrogen bonding and increased size, has a positive impact.

Compound 8 showed good in vitro potency (MIC = 0.5 μM), low citoxicity, and high selectivity over mammalian ATP synthesis. A generation of spontaneous mutants unambiguously identified ATP synthase as the molecular target of this class. Interestingly, squaramide showed no cross-resistance with BDQ in clinical isolates, suggesting a different binding mode. Computational and genetic studies supported this observation by predicting an additional interaction with subunit *a* of the ATP synthase. In this model, compound 8 binds at the interface between *a* and *c* subunits and blocks the rotation process, then preventing ATP synthesis. In vivo studies provided a proof-of-concept for this class of compounds. However, further medicinal chemistry optimization is needed to address pharmacokinetic liabilities and obtain potential preclinical candidates. 

#### 3.5.3. Miscellaneous Compounds

The growing interest in ATP synthase as a target and the emerging of BDQ resistance has resulted in a number of HTS and in silico screenings to identify additional options for ATP synthase inhibition and novel chemical entities for medicinal chemistry optimization, such as epigallocatechin [114], thiazolidones [115], and diaminoquinazolines [116]. 

**Figure 20:**
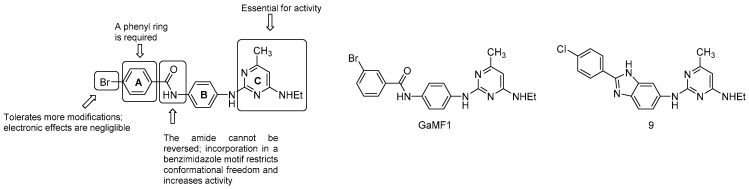
SAR analysis for GaMF class and chemical structures of GaMF1 and its improved analog 3.

A very recent work by Hotra et al. presented an interesting hit (GaMF1, Figure 20) of a novel class of derivatives targeting the γ loop of ATP synthase [117]. GaMF1 was identified through in silico screening. It showed a reasonable bactericidal activity (MIC = 33μM), both against sensitive and isoniazid and rifampicin-resistant strains, along with favorable ADME properties. A combination of biochemical, genetic, and NMR studies confirmed its target and revealed a synergistic effect but no cross-resistance to BDQ, raising the possibility of a new combination regimen. 

A series of analogues were synthesized to improve the antimycobacterial activity. SAR findings revealed the key features for activity (Figure 20):

- the C ring is essential;
- the amide link between the A and B ring cannot be reversed; however, the incorporation in a benzimidazole motif to restrict conformational freedom is possible and strongly increases potency (compound 9, MIC_50_ = 3 μM);
- the phenyl ring A is required but is more amenable to modifications: the electronics of the aromatic ring does not affect the potency.

The γ-loop is a unique target for developing novel ATP-synthase inhibitors: it is essential for ATP synthesis regulation, hydrolysis, and proton pumping, and lacks a homologue in humans and other prokaryotes [118]. Further studies and in vivo proof-of-concept of these inhibitors will shed light on their therapeutic potential and utility in TB treatment. 

### 3.6. Drugs Dissipating the Proton Motive Force (PMF)

High-density mutagenesis and deletion mutant studies showed that *Mtb* needs oxidative phosphorylation to grow [13]. In oxidative phosphorylation, energy generated due to the extrusion of protons across the biomembrane creates the PMF needed to drive ATP synthesis. As these mechanisms are essential for growth and survival in *Mtb*, developing small-molecule inhibitors which can break down the proton motive force is a valuable strategy to discover new anti-TB drugs. Several drugs have been shown to dissipate the PMF, not as the main mode of action. One of the most controversial is pyrazinamide (PZA). PZA, discovered in the early 1940s, is a frontline agent to treat TB [119]. PZA is a prodrug that in acidic conditions is hydrolyzed by pyrazinamidase to pyrazinoic acid (POA); indeed, most of the PZA clinical resistance arises from the loss of function mutations in *pncA* [120]. Multiple mechanisms of action and targets have been reported for PZA [120] including the dissipation of PMF, even if recent studies have established that PanD is the primary target of PZA [121].

#### SQ109

**Figure 21:**
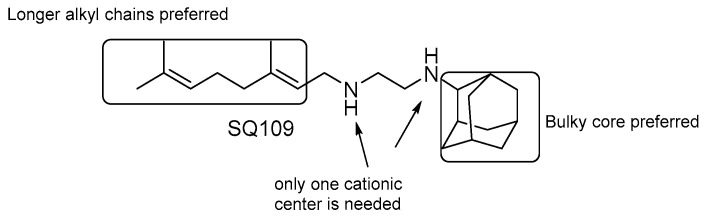
SAR analysis of SQ109.

SQ109 (Figure 21) is a 1,2-ethylenediamine-based compound developed by Sequella, Inc. in collaboration with Laboratory of Host Defenses, NIH [122]. A few clinical trials that have either been completed or are ongoing (Phase I in the US: NCT01358162 and NCT01585636, Phase 2 in South Africa: NCT01785186 and Phase 2b-3 study in Russia) showed favorable safety, satisfactory tolerability, and significant efficacy [123]. It was originally identified from a screening of a combinatorial library of ethambutol-based analogues. SAR analyses of several derivatives reveals that (Figure 21) [124,125]:

- the presence of two nitrogens is not essential for the activity, only one cationic center is needed [124];
- both size and nature of the alkyl substituents scaffold are critical for activity;
- highly α-branched aliphatic moieties are more effective.

Initially, it was proposed that SQ109 inhibits MmpL3 function, a *Mtb* membrane protein that transports trehalose monomycolate (TMM) into the cell envelope [126]. However, SQ109 is also active against organisms that lack a functional homolog of MmpL3, such as *Helicobacter pylori* [127], *Trypanosoma cruzi* [128], *Candida albicans* [122,129], and *P. falciparum* [124]. These observations, together with the evidence that no spontaneous *Mtb* resistant mutants were obtained, suggest that SQ109 might target multiple targets.

Indeed, further studies highlighted its ability to dissipate the PMF acting as an uncoupler, collapsing both ΔpH and Δψ, leading to a decrease in ATP synthesis [129]. Moreover, Li et al. have demonstrated that SQ109 and its analogues possess moderate activity against two enzymes of the menaquinone biosynthesis pathway, MenA and MenG [124].

### 3.7. SAR of Small Molecule Inhibitors Targeting CCM

*M. tuberculosis* spends most of its life inside the host cell in a state of slowed or arrested replication. Even as a non-replicating bacilli with minimal metabolic activity, *Mtb* has evolved unique metabolic adaptability to the host-imposed stringent niches in order to preserve its survival and ability to ensure transmission to a new host [130]. CCM provides energy and essential biosynthetic precursors, thus playing a key role in *Mtb* pathogenicity [131], and *Mtb* demonstrated the ability to use a plethora of organic substrates, such as carbohydrates, lipids, amino acids, and simple organic acids, to fuel it. While active *Mtb* requires carbohydrates as the main carbon source, *Mtb* in a latent phase that resides within the macrophage phagolysosome, where oxygen and nutrient depletion induce a massive metabolic rearrangement [132]. Notably, several analyses of *Mtb* transcriptional profiles from macrophages in vitro and from the lungs of mice and humans revealed a decreased glycolysis in concert with the upregulation of *Mtb* genes involved in lipids catabolism, proving that fatty acid ß-oxidation, gluconeogenesis, and cholesterol are the primary carbon and energy sources during infection in macrophages [133]. Despite the importance of CCM enzymes for promoting or maintaining a persistent infection, the presence of orthologous host enzymes could prevent the election of *Mtb* enzymes as potential drug targets. A remarkable exception is the enzymes involved in the glyoxylate shunt, a pathway required to shift from a reliance on carbohydrates to fatty acids as the main carbon source bypassing two steps of the TCA cycle [134].

#### 3.7.1. Inhibitors of Isocitrate Lyase

Two isoforms of ICL (ICL1: prokaryotic-like isoform and ICL2: eukaryotic-like isoform) have been described and are both required for *Mtb*’s survival and pathogenicity. The deletion of both *icl1* and *icl2* genes is necessary to induce the bacterial clearance from the host lung [135]. However, current research is mainly targeted at ICL1, as during infection the expression of *icl1* is one of the most highly upregulated genes. Additionally, the absence of ICL genes in mammals provides specificity, confirming the potential of ICL as a target for the development of safe drugs active against slow or non-replicating mycobacteria. However, the druggability of ICL has been a challenge mostly owing to its highly polar and small active site [11]. 

##### Phthalazinyl Hydrazones and Phthalazin-4-ylacetamide Derivatives

**Figure 22:**
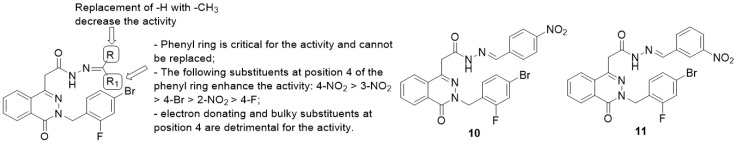
SARs of phthalazinyl hydrazones and chemical structures of compounds 10 and 11.

In the field of ICL inhibitors, Siram and co-workers [136] evaluated the antimycobacterial activity of phthalazinyl hydrazones and phthalazin-4-ylacetamides derivatives, designed as *Mtb* ICL inhibitors. With respect to the phthalazinyl hydrazones series, substitutions on the terminal acid hydrazone furnished benzaldehyde derivatives that exhibited higher activity than benzophenone and acetophenone analogs. Around the benzaldehyde scaffold, several SAR studies have been performed to ameliorate potency against the target enzyme, which can be summarized as follows (Figure 22):

- the phenyl ring is fundamental for the activity while the replacement with heteroaryls, such as the furanyl ring, has a negative impact;
- introduction of electron-withdrawing groups at position 4 of the phenyl ring provided the highest enhancement of the activity (4-NO_2_ > 3-NO_2_ > 2-NO_2_), particularly with the insertion of halogens and nitro groups (4-NO_2_ > 4-Br > 4-F);
- electron-donating groups (methyl, hydroxyl, methoxyl and dimethylamino substituents) and bulky groups at position 4 of the phenyl ring are detrimental for the activity;
- substitution of -R with methyl group provided less-active compounds.

Compound 10 was found to be the most active in vitro against log-phase cultures of both *Mtb* (MIC = 0.18 μM) and MDR-*Mtb* (MIC <0.09 μM), also showing a favorable safety profile with toxicity against Vero cell lines only above 122.5 μM, even better than the first-line anti-TB drugs INH and RIF (MICs of 0.66 μM and 0.23 μM, respectively) and to INH and ciprofloxacin against MDR-*Mtb* (MICs of 37.68 μM and 45.57 μM respectively). Isocitrate lyase inhibition studies have been executed using 3-NP (3-nitropropionate) as a standard ICL inhibitor. Compound 11 exhibited the highest inhibition rate towards ICL (66.70%) at 10 μM if compared to 3-NP, which furnishes 63.2% of inhibition at 100 μM. Compound 10 was tested in vivo against *Mtb* infected mice at 25 mg ⁄ kg but showed reduced anti-TB activity in lung and spleen (log CFU values of 6.12 and 5.99, respectively) compared to INH at the same dose level (log CFU values of 5.86 and 4.71 respectively). Further development efforts focused on the phthalazin core led to the synthesis of a series of phthalazin-4-ylacetamides, screened against log- and starved-phase *Mtb* [136].

**Figure 23:**
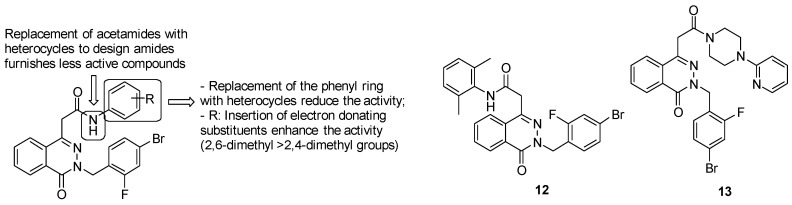
SARs of phthalazinyl acetamides and chemical structures of compounds 12 and 13.

Several manipulations on the aromatic ring have been performed to enhance bactericidal activity, as described in the following SARs (Figure 23):

- acetamide derivatives substituted with the phenyl ring provide high activity, while replacement of acetamide moiety with heterocycles led to less active compounds;
- replacement of the phenyl ring with another ring system, such as the pyridyl one, reduces the activity;
- insertion of electron donating groups, such as methyl one, on the ring strongly enhances the antimycobacterial activity.

Among phthalazin-4-ylacetamides, compound 12 was the most active in vitro against log-phase cultures of both *Mtb* (MIC = 0.18 μM) and MDR-*Mtb* (MIC = 0.08 μM), showing greater MIC values than INH, RIF and ciprofloxacin. Moreover, in a starved culture of *Mtb*, compound 12 was more active (MIC = 3.78 μM) than RIF (MIC = 15.2 μM). It also showed a favorable safety profile, with toxicity in Vero cell lines only above 126.43 μM. With respect to the enzyme target, compound 13 furnishes the highest rate of ICL inhibition (66.70%) at 10 μM. Compound 12 was assayed in vivo against *Mtb* infected mice at 25 mg⁄kg and unfortunately showed diminished anti-TB activity in the lung and spleen (log CFU values of 6.61 and 6.12, respectively) compared to INH (log CFU values of 5.86 and 4.71, respectively). 

##### Salycilanilides

**Figure 24:**
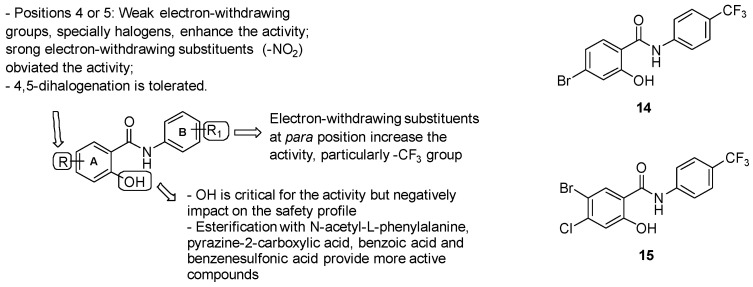
The SARs of salicylanilides and the chemical structures of compounds 14 and 15.

In 2003, Waisser and colleagues [137] identified salycilanilide derivatives as promising antimycobacterial agents, encouraging several research groups to extensively explore the SARs and the biological activities of this series. Various manipulations have been done around the 2 structural units (Figure 24):

- ring A: substitution with weak electron-withdrawing groups, particularly halogens, is preferred at positions 4 or 5, while strong electron-withdrawing substituents at the same positions (-NO_2_) affected the activity; notably, bromine derivatives result in being more active than chlorine analogues, likely due to the higher ClogP values of the final molecules bearing a bromine atom. The free phenolic hydroxyl result was essential for the activity but negatively impacted the safety profile;
- Aniline unit (ring B): the introduction of electron-withdrawing and lipophilic substituents enhanced the activity, with the best results provided by the trifluoromethyl group at position 4.

The most active salicylanilide described was compound 14 (Figure 24), with a MIC of 1 μM. Salicylanilides surprisingly demonstrated activity against MDR strains at lower values (0.5–4 μM) than those for drug-sensitive strains. Notwithstanding, salicylanilides endowed a discouraging cytotoxicity, since their IC_50_ values were comparable to their MICs, except for 4-bromo-*N*-(3,4-dichlorophenyl)-2- hydroxybenzamide and compound 14, which exhibited a more favorable profile. Thus, compound 14 furnished the best results in terms of activity and safety among the preliminary salicylanilide derivatives. More elucidation about the mode of action has been done by a work by Kratky M et al. [138], in which a new set of salicylanilide esters have been explored. Esterification with *N*-acetyl-L-phenylalanine, pyrazine-2-carboxylic acid, benzoic acid, and benzenesulfonic acid provided analogues with lower MIC values (ranging from 0.25 to 2 μM) than the parent salicylanilides (MICs = 1–8 μM). Among these derivatives, *N*-acetyl-L-phenylalanine esters furnished the best MICs (0.25-0.5 μM). Biological studies revealed that salicylanilides exert their antimycobacterial properties by disrupting multiple pathways, including methionine aminopeptidases and *Mtb* isocitrate lyase. When evaluated for their ICL1 inhibitory activity at 10 and 100 μM, salicylanilides and their 4-trifluoromethyl substituted esters showed the best inhibition rates at 10 and 100 μM (22% and 59%, respectively), comparable to those of 3-NP (25% and 67%, respectively). Lastly, a recent work by Paraskevopoulos et al. [139] highlighted the effects of two weak electron-withdrawing substituents (Cl and Br) at positions 4 and 5 of the salicylic part on the overall salicylanilides profile. Even if the introduction of the second halogen impacted the ClogP values, the 4,5-dihalogenated salicylanilides shared a similar antimycobacterial activity with the monohalogenated parental compounds, furnishing MIC values in the range of 1–4 μM. However, the dihalogenation mildly decreased the activity against MDR-Mtb and XDR-Mtb in comparison to the monohalogented analogs, ranging between 1–2 μM. Notably, dihalogenated molecules demonstrated diminished cytotoxicity, providing a more suitable profile for drugs. Compound 15 (Figure 23) emerged as the best of the series in terms of both activity (MIC = 1 μM) and safety.

#### 3.7.2. Inhibitors of Malate Synthase

Led by the noteworthy relevance of the glyoxylate shunt as an attractive target for tuberculosis therapeutics, potential inhibitors of malate synthase (GlcB) have been explored. GlbB furnishes a larger and more druggable active site than *Mtb* ICL, which naturally accommodates the pantothenate tail of the acetyl-CoA [140]. The presence of the catalytic Mg^2+^ persuaded the research community to assess a library of small Mg^2+^-dependent enzyme inhibitors with glyoxylate-like structures [141].

##### Phenyl-diketo Acids

**Figure 25:**
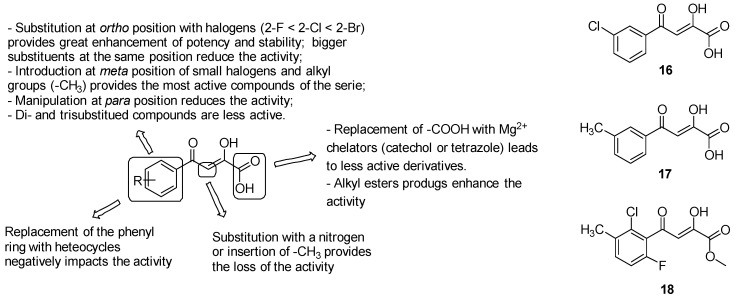
SARs of PDKAs and chemical structures of compounds 16, 17 and 18.

Among a library of 35 small glyoxylate-like molecules assayed against GlcB by Kriger et al. [142], phenyl-diketo acids (PDKAs) emerged as the most encouraging GlcB inhibitors. Consistent with these findings, a series of almost 100 PDKAs has been synthesized, providing IC_50_ values against the target enzyme ranging from 20 nM and >100 mM. Guided by the crystal structure of GlcB bound with the parent PDKA, SARs have been performed to ameliorate PDKAs activity and potency (Figure 25):

- the oxygens of the diketo acid moiety coordinate Mg^2+^ and provide hydrogen bonds with Asp462, Leu461, and Asp633, thus removing the diketo acid moiety generated inactive derivatives;
- modifications on the ß-carbon of the diketo acid as substitution with a nitrogen or insertion of a methyl group resulted in the loss of the activity;
- the replacement of the carboxylic acid with well-known Mg^2+^ chelators, such as catechol or bioisosteres as tetrazole, led to less-active derivatives;
- the aromatic ring is strongly required to establish both van der Waals interactions with Asp633, Met515, Trp541, Met63 and anion-π interactions [143] with the carboxylate of Asp633 side chain that is deprotonated during the catalyst; alternative structures including aliphatic cycles or heterocycles (naphthyl, indole, pyrrole, thiophene, furan, quinoline, benzodioxole, benzothiazole, thiazole, pyridine and pyrimidine) fail to provide a generally favourable combination of inhibitory activity against GlcB and pharmacokinetics;
- *o*-substituted analogues with small groups, especially halogens (2-F < 2-Cl < 2-Br), provided a great enhancement of potency, while the introduction of bigger or double substituents reduces the activity, probably due to steric interference with the Val118 side chain. Substitutions at the *ortho* position induced the twist of the phenyl ring out of plane and therefore increased stability by diminishing the degree of conjugation, which is responsible for the retro-Claisen decomposition of unsubstituted PDKAs in in various buffer solutions and cell growth media;
- manipulations at position 4 of the phenyl ring are less effective on the activity due to the undesirable steric clash of the substituents with the Met631 side chain;
- the introduction of substituents at the *meta* position to extend the parental PDKA structure afforded the best activity and potency by accommodating additional interactions in the acetyl-CoA binding channel, including that of the hydrogen bond with Val119 and van der Waals interactions with Met631, Met515, and Val118-Val119. Notably, the best results have been collected by the introduction of small halogens, such as fluorine, and alkyl groups such as methyl; bigger alkyl and aryl substituents negatively impact the activity, likely due to the steric interference with Met631 and Val118;
- di- and trisubstituted compounds such as 2-Cl-6-F-PDKAs are less active than monosubstituted PDKAs.

Consistent with these findings, the most active PDKAs derivatives were 3-Cl-PDKA (16) and 3-Me-PDKA (17) (IC_50_ = 0.17 mM and 0.18 mM, respectively, Figure 25).

Lastly, to ameliorate the limited cellular uptake of the carboxylic acid-bearing molecules, alkyl ester prodrugs of the most active PDKAs have been developed. This strategy remarkably impacted the antimycobacterial activity, providing lower MIC values compared to the parent PDKAs. The methyl ester 18 (Figure 25) showed the best results in terms of both potency and in vitro PK properties among the PDKAs series, providing efficacy in vivo in a mouse model of infection.

#### 3.7.3. Inhibitors of Cholesterol Catabolism

Cholesterol catabolism exerts a dominant role on *Mtb* physiology in more ways, such as being a fundamental source for both carbon and energy and influencing the CCM and various biosynthetic pathways. Particularly, cholesterol degradation furnishes pyruvate, acetyl-CoA, and propionyl-CoA, all of which sustain the CCM [144]. This evidence corroborates the importance of cholesterol catabolism for the bacterial persistence and growth inside the host macrophages and emphasize this pathway as a potential target to eradicate *Mtb* infection [145].

##### Azasteroids

Several series of azasteroids have been investigated as potential inhibitors of 3b-hydroxysteroid dehydrogenase (3b-HSD), an enzyme involved in sterol metabolism and required for the conversion of cholesterol to cholest-4-en-3-one [146]. Mycobacterial 3b-HSD shares low amino acid sequence homology of non-catalytic residues with human 3b-HSD, but the residues of both the active site and the binding motif with the NAD^+^ cofactor are conserved between orthologues; thus, selectivity for 3b-HSD cannot be guaranteed. 

**Figure 26:**
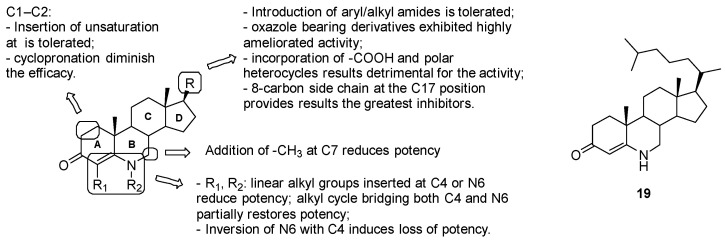
SARs of 6-azasteroids and the chemical structure of compound 19.

Several studies have been conducted on 6-azasteroids, transition state inhibitors of the 3b-HSD-catalyzed reaction with excellent PK properties in humans. Manipulations have been done on the 6-azasteroid core to explore the impact of substitutions at positions 17 of the D ring, 4 and 7 of the A-B rings, 1 and 2 of the A ring (Figure 26) [147]:

- position C17: the introduction of aryl/alkyl amides was tolerated; oxazole-bearing derivatives exhibited highly ameliorated activity, while the incorporation of carboxylic acid and polar heterocycles was detrimental for the activity; compound 19 with the hydrophobic 8-carbon side-chain at the C17 position, which mostly mimics the cholestenone substrate, proved to be the greatest at binding competitive inhibitors;
- substitutions at C1, C2, C7, C4 and N6 on the A-B ring system are poorly effective due to the stringent steric requirements for effective interaction with the enzyme.

In conclusion, inhibition studies revealed the promising activity of compound 19, which exhibited competitive inhibition of the target enzyme by blocking DHEA and uncompetitive inhibition with respect to NAD+ (IC50 against *Mtb* 3b-HSD: 0.5 ± 0.1 μM).

### 3.8. Inhibitors of PrpC (Methyl Citrate Cycle)

The cholesterol metabolic pathway induces metabolic toxicity associated with the excess of propionyl-CoA that the bacterium cannot assimilate [148]. To minimize this toxicity, *Mtb* makes drastic metabolic rearrangements, such as the use of propionyl-CoA to fuel the lipids biosynthesis and synthesize methyl-branched polyketide lipids such as phthiocerol dimycocerosate (PDIM), a virulence-associated lipid [144]. In this scenario, the 2-methylcitrate synthase (PrpC or 2-MC) represents a critical enzyme of the methyl citrate cycle (MCC), required in the assimilation of propionyl-CoA by the condensation of oxaloacetate with propionyl-CoA to form 2-methylcitrate. Blocking this pathway induces high sensitivity to propionate, which acts as a poison and reduces virulence. Therefore, PrpC has been identified as an attractive target for new anti-TP agents [149]. Preliminary studies identified Compound V-13–009920 as a promising PrpC inhibitor, able to prevent *Mtb* survival in cholesterol-containing media; however, more work is needed to define the precise role and in vitro and in vivo activities [150].

## 4. Conclusions

The urgent need to discover new and more effective medicines to combat TB has prompted the discovery of new targets. With the approval of bedaquiline, which targets mycobacterial energy production [12], and delamanid, which targets cell wall synthesis and energy production, the energy-metabolism in *Mtb* [12] has received much attention in the last decade as a potential target to investigate for the development of new antimycobacterial drugs. Moreover, in addition to the antimycobacterial activity exerted by ETC inhibition, it has been demonstrated that a secondary effect of targeting this pathway would be a reduction in the activity of the many PMF/ATP-dependent efflux pumps (EPs) responsible of drug resistance. Indeed, EPs are postulated to be dependent on the PMF and the presence of sufficient ATP concentrations within the cell. Thus, indirect EPs inhibition should lead to higher intracellular concentrations of drugs and accelerate cell death [151]. 

The vigorous search for new entities targeting mycobacterial energy production led to the identification of several drug candidates, the most advanced of which are the imidazopyridine Q203, the clofazimine analogue TBI-166 and the bedaquiline analogue TBAJ-876 [2]. To date, it has been demonstrated that a triple combination of BDQ, Q203, and clofazimine efficiently killed *Mtb* in vitro and in a macrophage model [152]. This may open up to new possibilities for novel combination therapies, independently from the current frontline drugs.

## Figures and Tables

**Table 1:** clogP, hERG activity, antimycobacterial efficacies in vitro and *in vivo,* and IV mouse clearance for BDQ and its improved analog TBAJ-876.

Compound	clogP	hERG IC_50_ (μM)	Mtb MIC_90_ (μM)	Log_10_ CFU_reduction_	IV cl (mL/min/kg)
**Bedaquiline**	7.25	1.6	0.03	4.5–6.1	7
**TBAJ-876**	5.15	>30	0.004	>5.5	13
